# Stepwise evolution of the developmental and symbiotic functions of DELLA in land plants

**DOI:** 10.1111/nph.71597

**Published:** 2026-09-28

**Authors:** Katharina Melkonian, Tifenn Pellen, Katinka Bünger, Océane Thiercelin, Aurélie Le Ru, Mélanie K. Rich, Matheus E. Bianconi, Jean Keller, Maxime Bonhomme, Pierre‐Marc Delaux

**Affiliations:** ^1^ LRSV, CNRS, Toulouse INP, Université de Toulouse Castanet‐Tolosan 31320 France; ^2^ TRI‐FRAIB Imaging Platform Facilities FRAIB, CNRS, Université de Toulouse Castanet‐Tolosan 31320 France

**Keywords:** DELLA, evolution, gibberellin, GRAS, *Marchantia*, symbiosis

## Abstract

Proposed model for the stepwise evolution of DELLA functions in the green lineage.

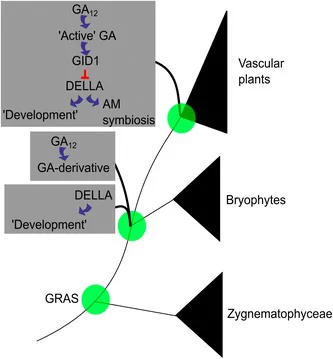

## Disclaimer

The New Phytologist Foundation remains neutral with regard to jurisdictional claims in maps and in any institutional affiliations.

## Introduction

In flowering plants, DELLA (named after a conserved amino acid motif containing Aspartate (D), Glutamate (E), Leucine (L), Leucine (L), and Alanine (A)) proteins are GIBBERELLIN INSENSITIVE, REPRESSOR OF GA1‐3, and SCARECROW (GRAS) transcription factors and play multiple functions mediated by the plant hormone gibberellin (GA). DELLA are master growth repressors and are subjected to degradation by the proteasome in the presence of bioactive GA (Dill *et al*., 2004). High‐yield dwarfed crop plants were found to have mutations in *DELLA* and to be less responsive to GA (Peng *et al*., 1999; Hedden, 2003). The GA‐induced degradation of DELLA proteins is mediated by the GA‐receptor GA INSENSITIVE DWARF1 (GID1) (Ueguchi‐Tanaka *et al*., 2005; Griffiths *et al*., 2007). DELLA proteins are also regulators of photomorphogenesis (Huq, 2002; Huq *et al*., 2004; De Lucas *et al*., 2008; Feng *et al*., 2008).

DELLA, GID1, and common bioactive GAs have been detected in all tested vascular plant lineages (Hirano *et al*., 2007; Yasumura *et al*., 2007; Yoshida *et al*., 2018). By contrast, both GID1 and common bioactive GAs are absent from bryophytes (Rensing *et al*., 2008; Bowman *et al*., 2017; Rich *et al*., 2021). Yet, knocking‐out *DELLA* in the moss *Physcomitrium patens* or overexpression of DELLA in the liverwort *Marchantia polymorpha* lead to developmental defects (Hernández‐García *et al*., 2021; Phokas *et al*., 2023). *Physcomitrium patens della* mutants are impaired in sporophyte development and display an increased spore germination rate (Phokas *et al*., 2023). *Marchantia polymorpha DELLA* ^*OE*^ lines are smaller than wild‐type plants, and overexpression of *DELLA* caused a loss of gemma dormancy. In addition, *M. polymorpha DELLA* ^*OE*^ lines are delayed in sexual reproduction (Hernández‐García *et al*., 2021).

In flowering plants, DELLA was also identified as a positive regulator of symbiosis establishment with diverse microorganisms, including arbuscular mycorrhizal (AM) fungi (Floss *et al*., 2013; Yu *et al*., 2014; Jin *et al*., 2016). For instance, in the legume *Medicago truncatula*, *della* mutants show decreased colonization by AM fungi and a lack or reduction in arbuscule formation (Floss *et al*., 2013; Foo *et al*., 2013; Jin *et al*., 2016). In rice, the effect is even more pronounced, with the almost total absence of colonization (Yu *et al*., 2014). Protein–protein interaction studies proposed that DELLA is part of a nuclear‐localized complex with components of the common symbiosis pathway, which is essential for symbiosis (Jin *et al*., 2016). Because the two model nonvascular plants *P. patens* and *M. polymorpha* have lost the ability to engage in the ancestral mutualistic symbiosis with AM fungi (Radhakrishnan *et al*., 2020), the evolutionary conservation of the symbiotic function of DELLA has never been investigated.

The liverwort *Marchantia paleacea* has become the model of choice to study the conservation of the AM symbiosis across land plants by comparison with vascular plants. So far, all the tested symbiotic mechanisms were found conserved across land plants, including the perception of fungal‐derived chemical signals (Tan *et al*., 2025; Teyssier *et al*., 2025), the integration of these signals by the common symbiosis pathway (Lam *et al*., 2024; Rich *et al*., 2025; Vernié *et al*., 2025), arbuscule formation (Sgroi *et al*., 2024), and the transfer of lipids to the fungal symbiont (Rich *et al*., 2021).

To investigate the evolutionary conservation of GA‐independent DELLA functions during development and AM symbiosis, we generated CRISPR/Cas9‐mediated *M. paleacea della* mutants, as well as double mutants (Mpa*della/gras13*) with its closely related *GRAS13* paralog. Phenotyping revealed that the origins of the developmental and arbuscule‐related symbiotic functions of DELLA were uncoupled during the evolution of land plants.

## Results and Discussion

### The single *DELLA* gene plays a developmental role in *M. paleacea*

To characterize the expression pattern of DELLA (Marpal_utg000095g0145961) in *M. paleacea*, we first generated *MpaDELLA*_*pro*_ *:GUS* lines and monitored the DELLA_pro_ activity. In mature thalli, blue staining from the GUS‐reporter was mainly observed in the photosynthetic layer, with a strong focus in assimilatory filaments at air pores (Fig. 1a), which may support a function of DELLA during photosynthesis regulation, and in other cases as well in rhizoids (Fig. 2a, Supporting Information Fig. S4, see later). To determine the potential roles of DELLA in development and AM symbiosis, we aimed at generating *M. paleacea della* mutants. Previous attempts in *M. polymorpha* did not allow generating viable *della* mutants, limiting the functional characterization to lines overexpressing *DELLA* (Hernández‐García *et al*., 2021). Using CRISPR/Cas9, we managed to generate five *della* mutants in *M. paleacea* (Figs 1b, S1a). Mutations ranged from single‐base substitutions to 62‐base deletions, leading to predicted premature stop codons for *della‐1*, *della‐3*, *della‐4*, and *della‐5* or an in‐frame substitution within the *DELLA* domain in case of *della‐2* (Fig. S1a). Because the growth of *M. paleacea* is not homogenous under axenic conditions on agar medium, we conducted the developmental phenotyping under nonaxenic conditions with plants grown on zeolite substrate in pots, similar to the promoter GUS conditions. We monitored the growth of *Mpadella* mutants over time, which revealed a significant growth retardation of *della* mutants compared to control plants at a light intensity of 50 μmol photons m^−2^ s^−1^ (Fig. 1c,d). We quantified the number of gemma cups after 57 d and found that all five *della* mutant alleles produced significantly fewer gemma cups than control plants (Fig. 1e). In addition, all five *della* mutant alleles appeared less green than control plants based on image quantification (Fig. 1f), and both Chl*a* and Chl*b* absorbance were significantly lower in four and three out of five mutant alleles compared to control plants, respectively (Fig. 1g).

**Fig. 1:**
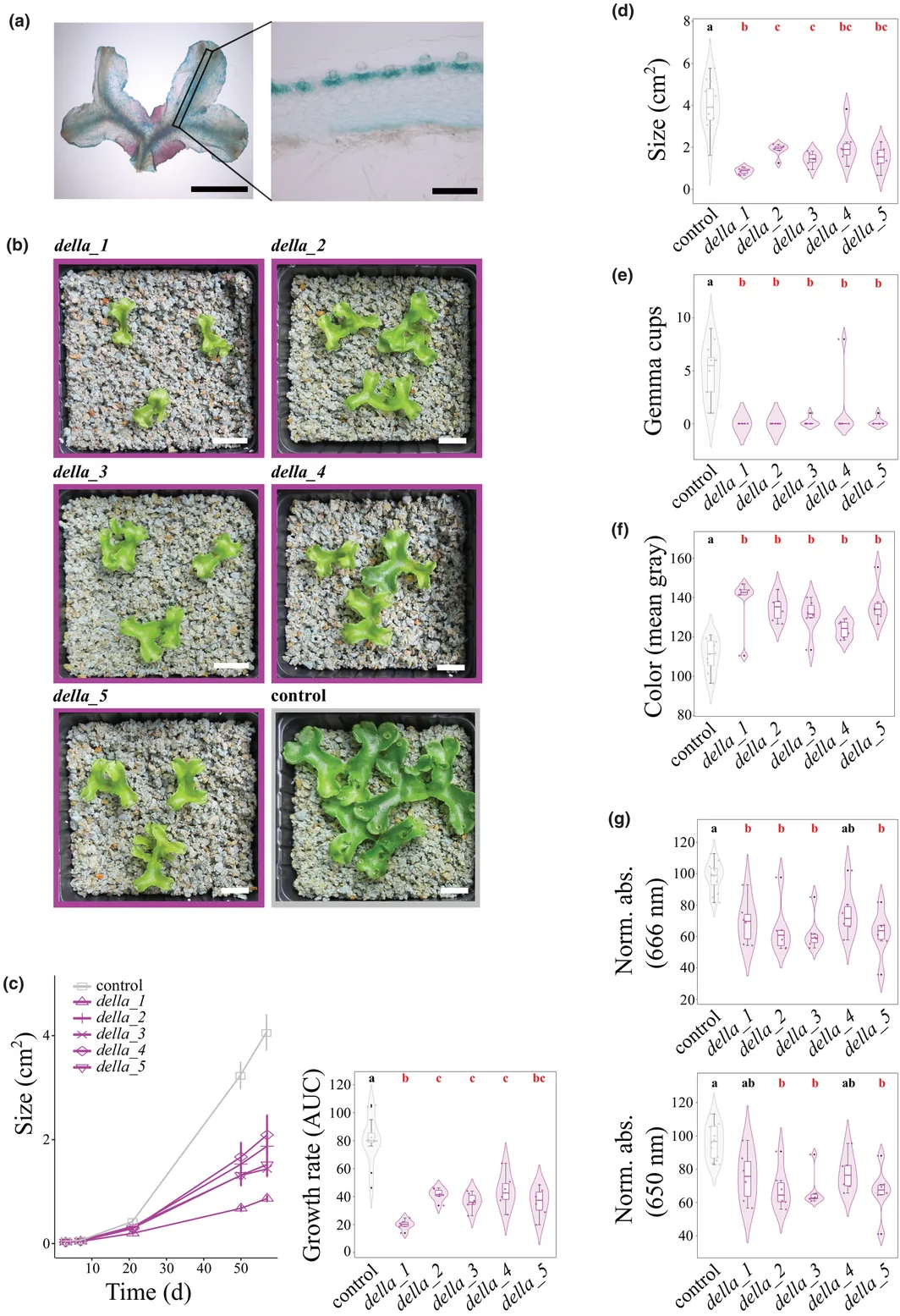
*Marchantia paleacea della* mutants are impaired in development. (a) Promoter‐GUS reporter expression in *MpaDELLA:GUSpro* lines at 57 d. Left: overview (bar, 1 cm). Right: longitudinal section (bar, 0.5 mm). (b) Representative overview pictures of *Mpadella* and control plants at 57 d, grown at 50 μmol photons m^−2^ s^−1^. Bars, 1 cm. (c) Thallus size quantification over the course of 57 d. Left: time course; data are mean ± SE (*n* = 6). Right: area under the curve (AUC) per plant; statistics were performed using pairwise Wilcox test and *P*‐values were corrected using the Bonferroni method (*n* = 6). (d) Thallus size at 57 d (*n* = 6). Statistics were performed using Welch's ANOVA with Games–Howell *post hoc* correction of *P*‐values. (e) Gemma cup number per genotype (*n* = 6). Statistics were performed using Kruskal–Wallis test and Dunn (BH) correction of *P*‐values. (f) Mean gray value of thallus area (*n* = 6). Statistics were performed using Welch's ANOVA with Games–Howell *post hoc* correction of *P*‐values. (g) Relative Chl*a* (upper) and Chl*b* (lower) absorption. Combined results of two independent experiments are shown (*n* = 6). Statistics were performed using Welch's ANOVA with Games–Howell *post hoc* correction of *P*‐values. Two independent repetitions of the whole experiment were performed. For boxplots in (c–g), the box indicates the middle 50% of the data, the line indicates the median value (50^th^ percentile), the bottom of the box represents the 25^th^ percentile (Q1), the top of the box represents the 75^th^ percentile (Q3), and the whiskers represent the interval between minimal and maximal values. Violin plots were generated using the Plöttr Toolbox (https://evompmi.github.io/plottr/).

**Fig. 2:**
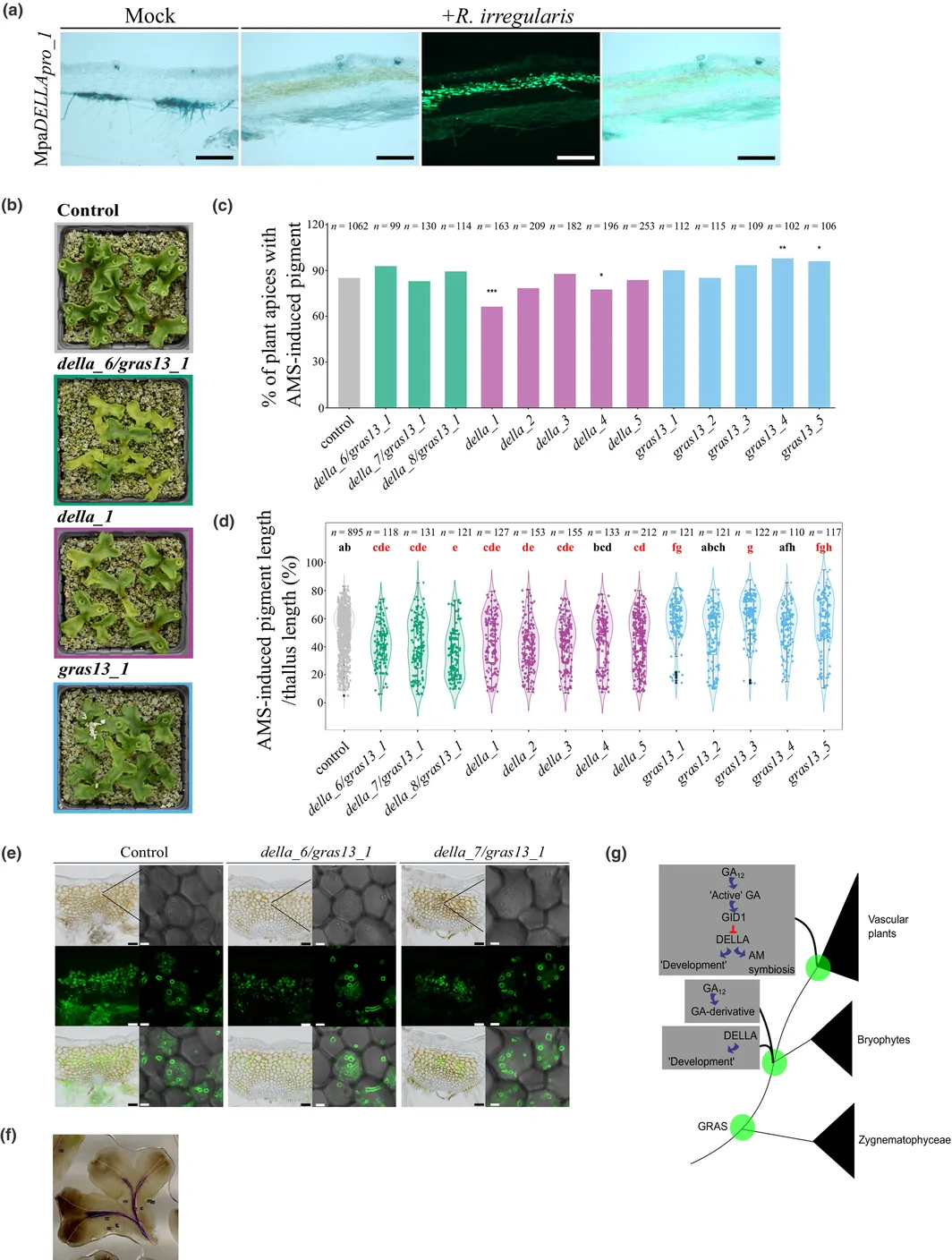
*Marchantia paleacea* DELLA is not essential for arbuscular mycorrhizal symbiosis. (a) Promoter activity of the *Marchantia paleacea DELLA* monitored using *MpaDELLApro:GUS* at 6 wk post inoculation. From left to right, longitudinal section of mock‐inoculated (bright field), *Rhizophagus irregularis*‐inoculated (bright field), *R. irregularis*‐inoculated (WGA‐Alexafluor 488), *R. irregularis*‐inoculated (merged) plants. (b) Representative images of control plants transformed with an empty vector, *Mpadella/gras13*, *Mpadella*, and *Mpagras13* plants inoculated with *R. irregularis* at 6 wk post inoculation. (c) Scoring of plant apexes with Arbuscular Mycorrhizal Symbiosis (AMS)‐induced pigment at 6 wk post inoculation with *R. irregularis* in control plants transformed with an empty vector and *Mpadella/gras13*, *Mpadella*, and *Mpagras13* mutants. Statistics were performed using pairwise Chi^2^ test with control plants (combined data of two independent lines) as the reference group, and *P*‐values were corrected using the Benjamini–Hochberg method. Combined results of six independent experiments are shown. (d) Progression of colonization measured as the ratio of the length of the pigmented midrib and thallus length (%) at 6 wk post inoculation with *R. irregularis* in control plants transformed with an empty vector and *Mpadella/gras13*, *Mpadella*, and *Mpagras13* mutants. Combined results of six independent experiments are shown. Statistics were performed using Kruskal–Wallis test and Dunn (BH) correction of *P*‐values. Red letters indicate statistically significant differences from control plants. For the violin plot, the box indicates the middle 50% of the data, the line indicates the median value (50^th^ percentile), the bottom of the box represents the 25^th^ percentile (Q1), the top of the box represents the 75^th^ percentile (Q3), and the whiskers represent the interval between minimal and maximal values. Violin plot was generated using the Plöttr Toolbox (https://evompmi.github.io/plottr/). (e) Representative images of control plants transformed with an empty vector and *Mpadella/gras13* double mutants inoculated with *R. irregularis* at 6 wk post inoculation. Green channel: WGA‐Alexafluor 488 staining of fungal structures. Bars, 100 μm (left panels) or 10 μm (right panels). (f) Example of measurements on mycorrhized thalli underlying results in (d). (g) Proposed model for the stepwise evolution of DELLA functions in the green lineage. GIBBERELLIN (GA) INSENSITIVE, REPRESSOR OF GA1‐3, and SCARECROW were gained in Zygnematophyceae via Horizontal Gene Transfer (HGT) (Cheng *et al*., 2019). In bryophytes, DELLA functions independently of GA and its precursors in development. GA‐dependent regulation of DELLA evolved in vascular plants and was co‐opted as an additional layer of control during arbuscular mycorrhizal symbiosis.

Overexpression experiments in *M. polymorpha* suggested that DELLA might play a role integrating growth and light responses (Hernández‐García *et al*., 2021). To further test the hypothesis that *della* mutants respond to different light regimes, control lines and *della* mutants were grown under elevated (75 μmol photons m^−2^ s^−1^) and high (100 μmol photons m^−2^ s^−1^) light regimes (Figs S2a,c, S3). In control lines, modifying light conditions resulted in an overall similar growth rate compared to normal light conditions (50 μmol photons m^−2^ s^−1^) for elevated light regime and a slightly increased growth speed at high‐light regime (Fig. S3a). By contrast, in *della* mutant plants, higher light intensities led to a stronger, but highly nonhomogenous increase in growth rate for the same plants, with asymmetric growth patterns of thalli (Figs S2a,c, S3a–c). This asymmetric growth effect was stronger in the elevated light regime, leading to an overall lower difference in growth rate over the course of 57 d (Fig. S3b) and in thallus size at 57 d of the *della* mutants (Fig. S3c). All five *della* mutants produced less gemma cups than control plants at all light regimes (Fig. S3d). A similar trend was observed for green coloration based on image quantification (Fig. S3e), and Chl*a* and Chl*b* absorption, although with some variability between alleles and light regimes (Fig. S3f). The expression pattern of *DELLA*, as per promoter‐GUS reporter lines, did not drastically change between different light regimes, but appeared slightly more focused around the midrib in air chambers at high‐light regime (Fig. S2b,d).

We propose that DELLA is a positive regulator of growth and gemma cup formation in *M. paleacea*, which are regulated in a light‐dependent manner to some extent and might be linked to Phytochrome‐interacting factors (PIF) signaling (Hernández‐García *et al*., 2021) and Chl biosynthesis.

### *MpaDELLA* is not required for the arbuscular mycorrhizal symbiosis

In flowering plants, mutations in *della* lead to severe symbiotic defects, including abolished or strongly reduced colonization, and the lack or strong reduction in arbuscule formation (Floss *et al*., 2013; Foo *et al*., 2013; Yu *et al*., 2014). To investigate potential symbiont‐dependent alterations in expression patterns, we first analyzed the *MpaDELLApro:GUS* lines following inoculation with the AM fungus *Rhizophagus irregularis* (Figs 2a, S4). Compared to the mock‐inoculated condition, an additional faint signal was observed in the colonized area of the thallus (Fig. 2a), while overall we did not observe an increased expression or changed pattern in seven independent *MpaDELLApro:GUS* transformant lines (Fig. S4). This is in line with RNAseq results that Mpa*DELLA* is not differentially regulated during AM symbiosis (Rich *et al*., 2021). To investigate the function of *DELLA* in *M. paleacea* during symbiosis, control plants and *della* mutants were inoculated with *R. irregularis*, and AM colonization was assessed after 6 wk. We scored the percentage of infected plants based on the presence of the AM symbiosis‐induced purple pigment as a proxy (Vernié *et al*., 2025). We found that two out of five mutant alleles displayed significantly reduced infection by *R. irregularis* (Fig. 2c). We propose that the discrepancy between mutant lines might be caused by variability in plant growth or differences in mutations (Fig. S1a), or loci of T‐DNA insertion. Thus, we quantified the progression of fungal colonization by normalizing the pigmented area by thallus length, to account for differences in thallus growth between *della* mutants and control plants (Fig. 2d,f). We found that fungal colonization was significantly less progressed in four out of five *della* mutant alleles compared to control plants (Fig. 2d). While we accounted for the differences in thallus size in this analysis, it should be interpreted with caution and might be the result of a reduced photosynthetic capacity of the *della* mutants compared to control plants. Finally, we analyzed the development of the fungal structures in the thalli. We found that arbuscules were present in all five *della* mutant alleles with no morphological impairments (Fig. S5b), which is in sharp contrast to the findings described in all tested model flowering plants (Floss *et al*., 2013; Jin *et al*., 2016).

We conclude from the analysis of the five *M. paleacea della* mutants that loss of a functional DELLA leads to a quantitative decrease in overall colonization that is independent of plant size, yet the formation of arbuscules is not impaired.

### *MpaDELLA* and its paralog *MpaGRAS13* are not redundant for AM symbiosis

The lack of a strong AM symbiosis defect similar to angiosperms in the *M. paleacea della* mutants might either indicate that symbiosis signaling differs between angiosperms and bryophytes or be the result of functional redundancy. Our phylogenetic analysis conducted on *DELLA* homologs confirmed the existence of a liverwort‐specific *DELLA* paralog (Hernández‐García *et al*., 2019), namely Mpa*GRAS13* (Marpal_utg000103g0151291), which likely evolved from a duplication in bryophytes followed by subsequent losses in mosses and hornworts (Fig. S6). To determine whether *M. paleacea GRAS13* could act redundantly with *DELLA*, we generated single *gras13* and *della/gras13* double mutants (Fig. S1), and phenotyped them in the presence of AM fungi. No strong developmental phenotypes were observed in the case of *gras13* single mutants, while the *della/gras13* double mutants displayed developmental phenotypes similar to the *della* mutants, but to a stronger extent (Figs 2b, S5a). In particular, *della/gras13* double mutants showed a strong asymmetric growth pattern and a complete absence of gemma cups in all three mutant alleles (Fig. S5a). To test whether this developmental defect might be due to the low‐phosphate condition in which the plants were grown (15 μM), we conducted an experiment under high phosphate conditions (2.5 mM phosphate). Although all genotypes were generally bigger, the asymmetric growth patterns and green coloration of the *della* and *della/gras13* mutants persisted (Fig. S7).

When scoring AM symbiosis using the AM symbiosis‐induced pigment, we found that two out of five *gras13* mutant alleles displayed a significantly increased percentage of infected plants compared to control plants (Fig. 2c). Next, we quantified the progression of fungal colonization in *gras13* single and *della/gras13* double mutants as described previously for the *della* mutants. Fungal colonization was significantly increased in three out of five *gras13* single mutant alleles, indicating a slight overcolonization, and was significantly delayed in all *della/gras13* double mutant alleles compared to control plants (Fig. 2d). Intracellular arbuscules were present in all *gras13* single and all *della/gras13* double mutant alleles, with no obvious morphological defects compared to control plants (Figs 2e, S5b). Finally, to investigate potential functional defects, we quantified the expression of symbiotic marker genes (*MpaRAM2C, MpaSTR, MpaSTR2*) by quantitative reverse transcription polymerase chain reaction in control plants and the *della*, *gras13*, or *della/gras13* mutant lines 5 wk post inoculation with *R. irregularis* (Fig. S8). We also included Rhizophagus irregularis Translation Elongation Factor 1 (RiTEF), a constitutively expressed fungal gene, as a proxy for colonization level. Given the rather early stage of symbiosis establishment, we found that fungal colonization based on Ri*TEF* quantification was variable for the different genotypes, including control plants (Fig. S8). This difference was mirrored by the expression of *RAM2C*, *STR*, and *STR2*, which covaried with *RiTEF* (Fig. S8).

We conclude from the analysis of the *M. paleacea della/gras13 double* mutants that *DELLA* mutation leads to a quantitative decrease in overall colonization, possibly due to affected growth, but not in arbuscule formation or symbiotic functionality. This is in sharp contrast with the major colonization and arbuscule‐formation defects observed in angiosperms. The milder symbiotic defect observed in *M. paleacea* is not due to genetic redundancy with the *DELLA* paralog *GRAS13*.

### A hypothetical model for the evolution of DELLA function in land plants

Biochemical surveys coupled with phylogenetic analyses of the GA biosynthesis and signaling pathways revealed that the evolution of DELLA predated the production of common bioactive GAs (Fig. 2g; Sun *et al*., 2023). Indeed, while DELLA and the upstream part of the biosynthesis, down to *ent‐kaurenoic acid* and the intermediate GA_12_ (Sun *et al*., 2023), are present in nonvascular plants, the GA receptor GID1 and GA sensing are specific to the vascular plants (Hirano *et al*., 2007). This points to the evolution of a GA‐GID1‐DELLA module at the base of vascular plants (Hirano *et al*., 2007; Sun *et al*., 2023).

The five *M. paleacea della* mutants generated in this study confirmed the developmental role of DELLA in *Marchantia* previously suggested by overexpression (Hernández‐García *et al*., 2021). Together with the developmental defect of the *P. patens della* mutants (Phokas *et al*., 2023), this indicates that DELLA plays a GA‐independent developmental role in nonvascular plants.

In contrast to the developmental function, the lack of a strong AM symbiosis defect in the *M. paleacea della* and *della/gras13* mutants suggests that DELLA may not be involved to the same extent in symbiosis in nonvascular plants. This result is intriguing on its own. So far, all the symbiotic genes identified in angiosperms and tested for their symbiotic functions revealed a conserved role in *M. paleacea* (Lam *et al*., 2024; Sgroi *et al*., 2024; Rich *et al*., 2025; Teyssier *et al*., 2025; Vernié *et al*., 2025), including components acting together with DELLA in angiosperms such as CYCLOPS and CALCIUM AND CALCIUM/CALMODULIN‐DEPENDENT PROTEIN KINASE (CCaMK) (Vernié *et al*., 2025).

It can be proposed that during the evolution of vascular plants, GA_12_‐modifying enzymes emerged, leading to the biosynthesis of common bioactive GAs. The existing DELLA‐regulated module was co‐opted by these GAs via the gain of a functional GID1 receptor (Hirano *et al*., 2007; Yasumura *et al*., 2007; Yoshida *et al*., 2018). Interestingly, the GA intermediate identified in *M. polymorpha*, GA_12_, is the form transported via the vasculature in angiosperms (Da Fonseca *et al*., 2015). Beyond symbiosis, systemic regulations allowing for long‐distance communication across organs and tissue were one of the key innovations for vascular plants to evolve (Wheeldon & Bennett, 2021). We propose the hypothesis that DELLA has evolved and been maintained in the vascular plant symbiotic signaling pathway not directly because of its positive role, but rather because its degradation by GA brings an additional, systemic control layer on symbiosis. Although this model is purely hypothetical, it is aligned with currently available data and our results, and provides testable hypotheses for the future.

## Materials and Methods

### Plasmid construction and stable transformation of *Marchantia paleacea*

Genetic constructs for CRISPR‐Cas9‐mediated targeted mutagenesis of Mpa*DELLA* (Marpal_utg000095g0145961) and Mpa*GRAS13* (Marpal_utg000103g0151291) and promoter‐reporter assays were generated using the Golden Gate molecular cloning system as previously described (Engler & Marillonnet, 2014; Patron *et al*., 2015; Rich *et al*., 2021). By default, we used a 2000‐bp fragment upstream of the Start Codon of MpaDELLA as promoter sequence. A list of guideRNA sequences, as well as Level 0, 1, and 2 constructs, is provided in Table S1. *Marchantia paleacea* Bertol. wild‐type plants were grown on Gamborgs B5 half‐strength medium (G5768; Sigma) pH 5.7, 1.4% bacteriological agar (1330; Euromedex, Souffelweyersheim, France) for 4–5 wk in a walk‐in growth chamber at 22°C under a 16 h : 8 h, light : dark cycle. For each transformation, 15–25 gemmalings were blended in a sterile 250‐ml stainless steel bowl (Waring, Stamford, CT, USA) in 10 ml of 0M51C medium (KNO_3_ 2 g l^−1^, NH_4_NO_3_ 0.4 g l^−1^, MgSO_4_ 7H_2_O 0.37 g l^−1^, CaCl_2_ 2H_2_O 0.3 g l^−1^, KH_2_PO_4_ 0.275 g l^−1^, L‐glutamine 0.3 g l^−1^, casamino‐acids 1 g l^−1^, Na_2_MoO_4_ 2H_2_O 0.25 mg l^−1^, CuSO_4_ 5H_2_O 0.025 mg l^−1^, CoCl_2_ 6H_2_O 0.025 mg l^−1^, ZnSO_4_ 7H_2_O 2 mg l^−1^, MnSO_4_ H_2_O 10 mg l^−1^, H_3_BO_3_ 3 mg l^−1^, KI 0.75 mg l^−1^, EDTA ferric sodium 36.7 mg l^−1^, myo‐inositol 100 mg l^−1^, nicotinic acid 1 mg l^−1^, pyridoxine HCl 1 mg l^−1^, and thiamine HCl 10 mg l^−1^). The plant fragments were cultivated in 250‐ml Erlenmeyer flasks containing 15 ml 0M51C for 3 d with shaking (200 rpm) at 22°C under a 16 h : 8 h, light : dark cycle. The plant fragments were subsequently inoculated with 100 μl *Agrobacterium tumefaciens* GV3101 harboring the respective plasmids from a saturated liquid culture and supplemented with acetosyringone to a final concentration of 100 μM. The plant fragments were cocultivated with *Agrobacteria* for 3 d with shaking (200 rpm) at 22°C under a 16 h : 8 h, light : dark cycle, washed three times with water, and plated on Gamborgs B5 half‐strength solid medium containing 200 mg l^−1^ amoxycilin (Levmentin; Laboratoires Delbert, Paris, France) and 10 mg l^−1^ hygromycin (Duchefa Biochimie, Montpellier, France). Transformants were selected after 3‐ to 5‐wk cultivation at 22°C under a 16 h : 8 h, light : dark cycle.

### Phenotyping of Mpa*della* mutants at different light regimes

Three *M. paleacea* plants were grown in two pots per genotype on zeolite substrate (50% fraction 1.0–2.5 mm, 50% fraction 0.5–1.0‐mm, Symbiom) at 22–24°C in a walk‐in growth chamber under long‐day conditions (16 h : 8 h, light : dark) at different light intensities (low, 50 μmol photons m^−2^ s^−1^; elevated, 75 μmol photons m^−2^ s^−1^; high, 100 μmol photons m^−2^ s^−1^). The plants were watered once per week with Long Ashton solution containing 15 μM phosphate and plant growth was monitored over the course of 57 d. Two independent repetitions of the experiment were performed. Plant size and mean gray values were quantified using ImageJ. Gemma cup number was quantified on Day 57 of the experiment. For Chl absorbance determination, three plants per genotype and condition were harvested and weighed on Day 57 of the experiment. Chl was sequentially extracted from the plant tissues in 2 ml DMSO at 65°C for 4 h in the dark. Chl absorbance in the supernatant was determined using a photometer (P3 UVisco; LC Instrument, Lisses, France) at a wavelength of 666 nm for Chl*a* and 650 nm for Chl*b*. Absorbance values were normalized according to plant fresh weight per sample, and relative values were calculated through division by the mean absorbance of control plants per sample. Raw counts and calculated values are provided in Table S2. All data were plotted using https://evompmi.github.io/plottr/ or R Studio (v.2025.05.0).

### *Marchantia paleacea* mycorrhiza formation tests, P status, and promoter‐GUS reporter assays

*Marchantia paleacea* plants were grown in pots on zeolite substrate (50% fraction 1.0–2.5 mm, 50% fraction 0.5–1.0‐mm, Symbiom) at 22–24°C in a walk‐in growth chamber under long‐day conditions (16 h : 8 h, light : dark) at 65 μmol photons m^−2^ s^−1^ for 2 wk. For mycorrhiza formation tests and promoter‐reporter assays, plants were watered once per week with Long Ashton solution containing 15 μM phosphate. Five plants per pot were then mock‐inoculated or inoculated with *c*. 1000 sterile spores of *R. irregularis* DAOM 197198 provided by Agronutrition (Labège, France; https://www.agronutrition.com/en/contact‐us/). At 6 wk post inoculation, the plants were harvested. For promoter‐GUS reporter assays, the plants were vacuum‐infiltrated with GUS buffer (phosphate buffer (0.1 M), EDTA (5 mM), K_3_Fe(CN)_6_ (0.5 mM), K_4_Fe(CN)_6_ (0.5 mM), X‐Gluc (0.25 mg ml^−1^; Euromedex), and H_2_O) for 20 min at 200 mbar directly after harvesting and then incubated at 37°C overnight in the dark. The GUS buffer was removed, and the plants were washed once with water. All plants were then submerged in 100% ethanol to extract Chl until transparency was achieved and then stored in aqueous solution containing EDTA (0.5 mM) until further processing. Cleared thalli were scanned and scored as previously described (Rich *et al*., 2021), based on the presence or absence of a mycorrhiza formation–induced purple pigment. Statistics were perfomed using Chi^2^ test, and *P*‐values were corrected using the Benjamini–Hochberg method. Progression of colonization was determined by measuring the length of the pigmented zone and calculating the ratio with the total thallus length (data displayed as % of the length of the midrib that is pigmented; Fig. 2b,f). The thalli were subsequently analyzed by microscopic imaging to confirm the presence of intracellular fungal structures. For P status experiments, plants were grown for 2 wk in ‘Low P’ condition (Long Ashton solution containing 15 μM phosphate) or ‘High P’ condition (Long Ashton solution containing 2.5 mM phosphate). Plants grown in ‘Low P’ condition were subsequently mock‐inoculated or inoculated with *c*. 1000 sterile spores of *R. irregularis* DAOM 197198 provided by Agronutrition (https://www.agronutrition.com/en/contact‐us/). Plant growth between all three conditions (‘Low P’, ‘Low P + *R. irregularis*’, ‘High P’) was monitored for another 5 wk post inoculation and plant size, as well as color (mean gray) were quantified using ImageJ on Day 48 as described for the experiment at different light regimes. All data were plotted using https://evompmi.github.io/plottr/ or R Studio (v.2025.05.0). *Rhizophagus irregularis*‐inoculated plants were subsequently harvested and frozen in liquid nitrogen for quantitative polymerase chain reaction (qPCR) analyses.

### qPCR analyses

RNAs of *M. paleacea* control plants, Mpa*della*, Mpa*gras13*, and Mpa*della/gras13* mutant lines were extracted using a Direct‐zol RNA MiniPrep Zymo kit according to the supplier's recommendation on *c*. 100 mg of ground frozen thalli. Reverse transcription was performed using M‐MLV (Promega) on 500 ng of RNA and qPCR was performed on 5× diluted cDNA in a BioRad CFX opus 384 thermocycler with SYBR Green (Sigma). Relative expression values were calculated using the reference genes *MpaCULLIN* and *MpaAPT2* (data correspond to $2^{-\Delta C_{T}}$ with Δ*C*_T_ = CTgene of interest‐CTaverageCT*CULLIN* + *APT2*) or Mpa*CULLIN* and Mpa*APT2* alone.

### Microscopic imaging

Bright‐field images of whole thalli in promoter‐GUS reporter assays were acquired using a Zeiss Axiozoom V16 equipped with objective ×0.5. To visualize arbuscules and tissue‐specific expression patterns, thalli were embedded in 7% agarose and 100 μM transversal or longitudinal sections were prepared using a Leica vt1000s vibratome. The sections were incubated overnight in 12% KOH at room temperature and rinsed three times with water, followed by an incubation in 1 μg ml^−1^ WGA‐Alexa Fluor 488 (Invitrogen) in Phosphate‐Buffered Saline (PBS) buffer overnight at 4°C in the dark. The sections were then imaged using a Zeiss Axiozoom V16 equipped with objective ×0.5 and a GFP‐like filter set (Zeiss 38 He filter set, ex:470/40, em: 525/50) to visualize WGA or using a Nikon Ti Eclipse inverted microscope equipped with a DS Ri2 camera and motorized XY stage. Large images of global sections were generated using the NISAR 4.3 scan large image module that allows multifield acquisition and image stitching. These images were acquired with a ×10/0.3 dry objective (0.73 pixel size) in brightfield and in fluorescence for WGA‐Alexa 488 staining using a GFP band‐pass filter set (ex: 472/30 nm, em:520/35 nm). Close‐up images were acquired using a Leica SP8 TCSPC confocal microscope and LAS X software with a ×25 water immersion objective (Fluotar VISIR 25×/0.95 WATER) at zoom ×5 (0.182 μm pixel size). WGA‐Alexa 488 staining of fungal structures was excited with the 488 nm laser line and fluorescence was recovered between 500 and 550 nm. Bright‐field images were also performed and merged. Images were processed with ImageJ.

### Phylogenetic analyses

We used Blast v2.15.0 (Camacho *et al*., 2009) to search for putative homologs of DELLA proteins in the genomes of 26 land plant species, including 14 vascular plants and 12 bryophytes (Table S3), using the protein sequences of *M. paleacea* as queries (*e‐value* = 10^−9^). Sequences were aligned using Mafft v.7.505 (Katoh & Standley, 2013) with the automatic mode (*‐‐auto*, *‐‐maxiterate* 20), and subsequently trimmed using trimAl v.1.4.1 (Capella‐Gutiérrez *et al*., 2009) to remove alignment columns with > 80% of gaps (*‐gt* = 0.2). A phylogenetic tree was then estimated using FastTree v.2.1.11 (Price *et al*., 2010) with the gamma model (*‐gamma*). This resulting tree was inspected to identify the ortholog group containing the query sequences, which was extracted and realigned with the option *‐‐maxiterate* set to 1000 to increase alignment accuracy, and trimmed to remove columns with more than 75% of gaps (*‐gt* 0.25). A maximum‐likelihood tree was then estimated with IQ‐Tree v.2.2.2.6 (Minh *et al*., 2020) using the standard model selection (*‐m TEST*), with branch support assessed using the SH‐like approximate likelihood test and ultrafast bootstrap, both with 1000 replicates.

## Competing interests

None declared.

## Author contributions

KM, KB, OT and ALR conducted and analyzed the experiments. TP, KM and MKR generated the genetic resources. JK and MEB conducted and analyzed phylogenies. KM and MB performed statistical analyses. P‐MD and KM conceived and coordinated the project. P‐MD and KM wrote the manuscript.

## Supporting information


**Fig. S1** Gene and protein models of Mpa*della*, Mpa*gras13*, and Mpa*della/gras13* mutants.
**Fig. S2** Overview of Mpa*della* mutants and promoter activity at elevated and high‐light regimes.
**Fig. S3** Phenotypic characterization of Mpa*della* mutants at different light regimes.
**Fig. S4** Overview of Mpa*DELLA* expression profiles in absence or presence of *Rhizophagus irregularis*.
**Fig. S5** Macroscopic and microscopic phenotypes of Mpa*della*, Mpa*gras13*, and Mpa*della/gras13* mutants.
**Fig. S6** Phylogenetic tree of DELLA.
**Fig. S7** Increased phosphate supply does not fully rescue the developmental phenotypes of Mpa*della* and Mpa*della/gras13* mutants.
**Fig. S8** Symbiosis marker gene expression in Mpa*della*, Mpa*gras13*, and Mpa*della/gras13* plants.

**Table S1** Plasmids, guideRNA sequences and qPCR primers.

**Table S2** Supplementary data for *Marchantia paleacea della* experiments at different light regimes.

**Table S3** List of land plant species genomes used for the phylogenetic analyses.
Please note: Wiley is not responsible for the content or functionality of any Supporting Information supplied by the authors. Any queries (other than missing material) should be directed to the *New Phytologist* Central Office.

## Data Availability

All data are provided in the manuscript and Table S2 for plant growth data.
