# Supplementary material for: Stepwise evolution of the developmental and symbiotic functions of DELLA in land plants
Source: New Phytol. 2026 Sep 28;252(5):1702–10. doi: 10.1111/nph.71597 (PMC13738681; doi:10.1111/nph.71597)
Supplement: Supplementary file 1 [file NPH-252-1702-s003.pdf]

New Phytologist Supporting Information

Article title: Stepwise evolution of the developmental and symbiotic functions of DELLA in land plants

Authors: Katharina Melkonian, Tifenn Pellen, Katinka Bünger, Océane Thiercelin, Aurélie Le Ru, Mélanie K. Rich, Matheus E. Bianconi, Jean Keller, Maxime Bonhomme, Pierre-Marc Delaux

Article acceptance date: 14 August 2026

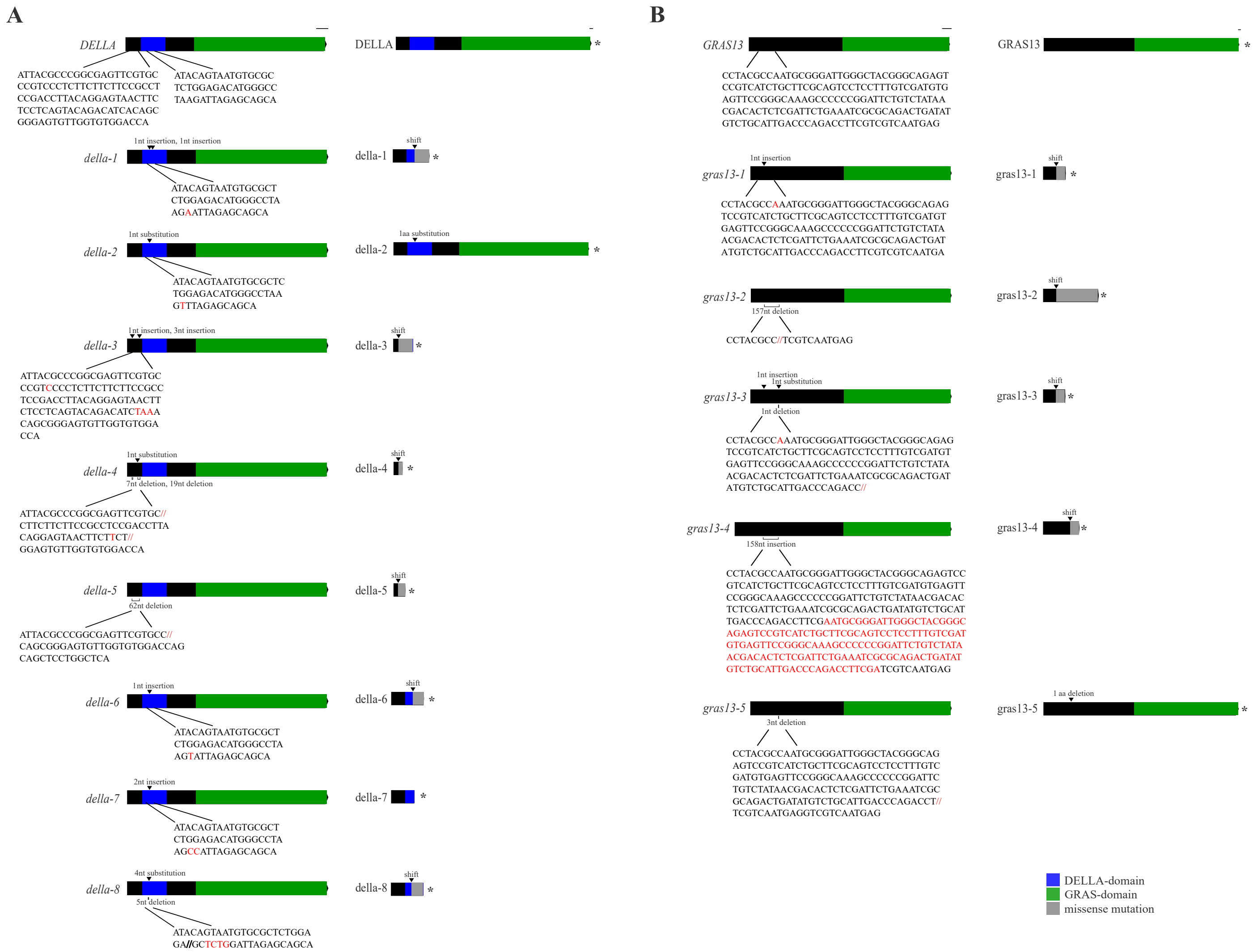

A
DELLA
ATTACGCCCGGCGAGTTCGTGC
CCGTCCCTCTTCTTCTTCCGCCT
CCGACCTTACAGGAGTAACTTC
TCCTCAGTACAGACATCACAGC
GGGAGTGTTGGTGTGGACCA
ATACAGTAATGTGCGC
TCTGGAGACATGGGCC
TAAGATTAGAGCAGCA
DELLA
*
1nt insertion, 1nt insertion
della-1
ATACAGTAATGTGCGCT
CTGGAGACATGGGCCTA
AGAATTAGAGCAGCA
della-1
shift
*
1nt substitution
della-2
ATACAGTAATGTGCGCTC
TGGAGACATGGGCCTAA
GTTTAGAGCAGCA
1aa substitution
della-2
*
1nt insertion, 3nt insertion
della-3
ATTACGCCCGGCGAGTTCGTGC
CCGTCCCTCTTCTTCTTCCGCC
TCCGACCTTACAGGAGTAACTT
CTCCTCAGTACAGACATCTAAA
CAGCGGGAGTGTTGGTGTGGA
CCA
shift
della-3
*
1nt substitution
della-4
7nt deletion, 19nt deletion
ATTACGCCCGGCGAGTTCGTGC//
CTTCTTCTTCCGCCTCCGACCTTA
CAGGAGTAACTTCTTCT//
GGAGTGTTGGTGTGGACCA
shift
della-4
*
della-5
62nt deletion
ATTACGCCCGGCGAGTTCGTGCC//
CAGCGGGAGTGTTGGTGTGGACCAG
CAGCTCCTGGCTCA
shift
della-5
*
1nt insertion
della-6
ATACAGTAATGTGCGCT
CTGGAGACATGGGCCTA
AGTATTAGAGCAGCA
shift
della-6
*
2nt insertion
della-7
ATACAGTAATGTGCGCT
CTGGAGACATGGGCCTA
AGCCATTAGAGCAGCA
della-7
*
4nt substitution
della-8
5nt deletion
ATACAGTAATGTGCGCTCTGGA
GA//GCTCTGGATTAGAGCAGCA
shift
della-8
*
B
GRAS13
CCTACGCCAATGCGGGATTGGGCTACGGGCAGAGT
CCGTCATCTGCTTCGCAGTCCTCCTTTGTCGATGTG
AGTTCCGGGCAAAGCCCCCCGGATTCTGTCTATAA
CGACACTCTCGATTCTGAAATCGCGCAGACTGATAT
GTCTGCATTGACCCAGACCTTCGTCGTCAATGAG
GRAS13
*
1nt insertion
gras13-1
CCTACGCCAAATGCGGGATTGGGCTACGGGCAGAG
TCCGTCATCTGCTTCGCAGTCCTCCTTTGTCGATGT
GAGTTCCGGGCAAAGCCCCCCGGATTCTGTCTATA
ACGACACTCTCGATTCTGAAATCGCGCAGACTGAT
ATGTCTGCATTGACCCAGACCTTCGTCGTCAATGA
shift
gras13-1
*
gras13-2
157nt deletion
CCTACGCC//TCGTCAATGAG
shift
gras13-2
*
1nt insertion
1nt substitution
gras13-3
1nt deletion
CCTACGCCAAATGCGGGATTGGGCTACGGGCAGAG
TCCGTCATCTGCTTCGCAGTCCTCCTTTGTCGATGT
GAGTTCCGGGCAAAGCCCCCCGGATTCTGTCTATA
ACGACACTCTCGATTCTGAAATCGCGCAGACTGAT
ATGTCTGCATTGACCCAGACC//
shift
gras13-3
*
gras13-4
158nt insertion
CCTACGCCAATGCGGGATTGGGCTACGGGCAGAGTCC
GTCATCTGCTTCGCAGTCCTCCTTTGTCGATGTGAGTT
CCGGGCAAAGCCCCCCGGATTCTGTCTATAACGACAC
TCTCGATTCTGAAATCGCGCAGACTGATATGTCTGCAT
TGACCCAGACCTTCGAATGCGGGATTGGGCTACGGGC
AGAGTCCGTCATCTGCTTCGCAGTCCTCCTTTGTCGAT
GTGAGTTCCGGGCAAAGCCCCCCGGATTCTGTCTATA
ACGACACTCTCGATTCTGAAATCGCGCAGACTGATAT
GTCTGCATTGACCCAGACCTTCGATCGTCAATGAG
shift
gras13-4
*
gras13-5
3nt deletion
CCTACGCCAATGCGGGATTGGGCTACGGGCAG
AGTCCGTCATCTGCTTCGCAGTCCTCCTTTGTC
GATGTGAGTTCCGGGCAAAGCCCCCCGGATTC
TGTCTATAACGACACTCTCGATTCTGAAATCGC
GCAGACTGATATGTCTGCATTGACCCAGACCT//
TCGTCAATGAGGTCGTCAATGAG
1 aa deletion
gras13-5
*
DELLA-domain
GRAS-domain
missense mutation

Fig S1. Gene and protein models of Mpa*della*, Mpa*gras13* and Mpa*della/gras13* mutants. **A.** Schematic overview of mRNA structure (left; scalebar: 100 basepairs) and predicted proteins (right) of wildtype Mpa*DELLA* and mutated versions (*della-1* to *della-5* display mutated sequences in the Mpa*della* single mutants, *della-6* to *della-8* display mutated sequences in the three Mpa*della*/Mpa*gras13* double mutants). Black arrows indicate the sites of mutations, mutations are highlighted in red, stars indicate predicted stop codons. **B.** Schematic overview of mRNA structure (left; scalebar: 100 basepairs) and predicted proteins (right; scalebar: 10 amino acids) of wildtype Mpa*GRAS13* and mutated versions (*gras13-1* to *gras13-5* display mutated sequences in the Mpa*gras13* single mutants, all three Mpa*della*/Mpa*gras13* double mutants carry the *gras13-1* mutation). Black arrows indicate the sites of mutations, mutations are highlighted in red, stars indicate predicted stop codons. Gene and protein models were generated with http://wormweb.org/exonintron and processed in Inkscape.

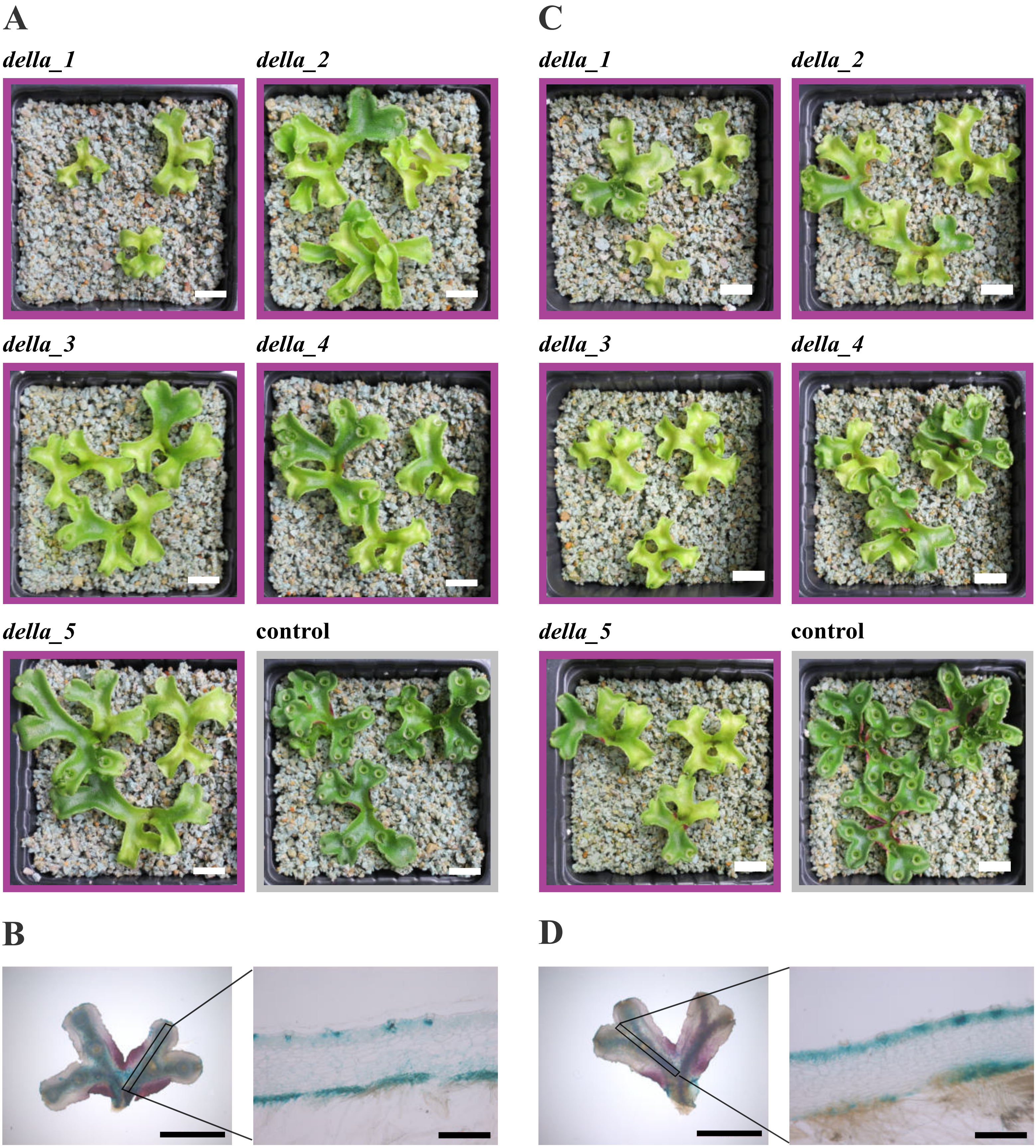
A
della_1
della_2
della_3
della_4
della_5
control
C
della_1
della_2
della_3
della_4
della_5
control
B
D

Fig S2. Overview of Mpa*della* mutants and promoter activity at elevated and high light regimes. Representative overview pictures of Mpa*della* and control plants at 57-days old, grown at **A.** 75 or **C.** 100 µmol photons m^{-2} s^{-1}. Scalebar: 1cm. Two independent repetitions of the experiment were performed. Promoter-GUS reporter expression in proMpa*DELLA:GUS* lines at 57-days old at **B.** 75 or **D.** 100 µmol photons m^{-2} s^{-1}. Left: Overview, scalebar: 1 cm. Right: Longitudinal section, scalebar: 0.5 mm.

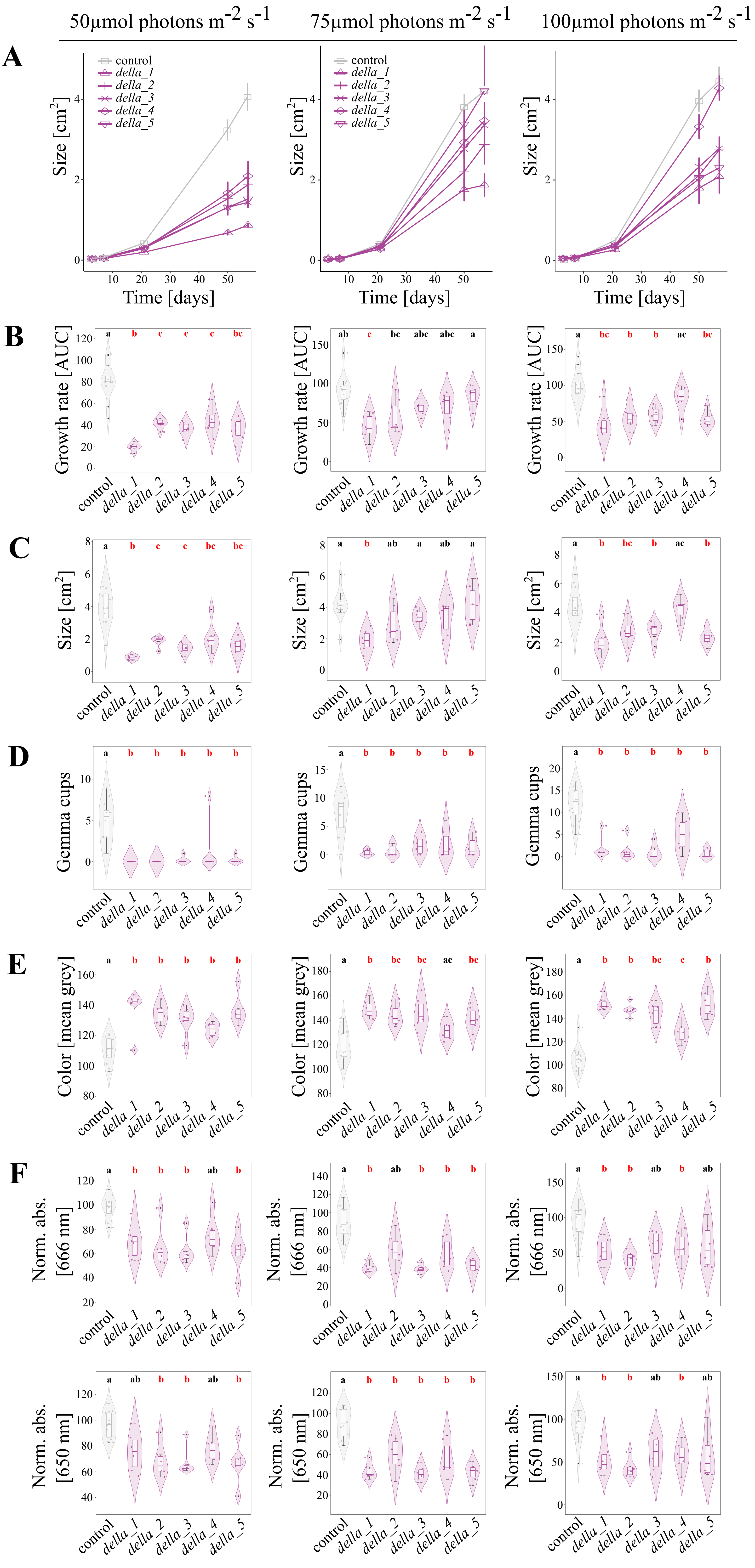
50µmol photons m-2 s-1
75µmol photons m-2 s-1
100µmol photons m-2 s-1
A
B
C
D
E
F
control
della_1
della_2
della_3
della_4
della_5
Size [cm2]
Time [days]
Growth rate [AUC]
Gemma cups
Color [mean grey]
Norm. abs. [666 nm]
Norm. abs. [650 nm]

Fig S3. Phenotypic characterization of Mpa*della* mutants at different light regimes.

Left: 50 µmol photons m^{-2} s^{-1}. Middle: 75 µmol photons m^{-2} s^{-1}. Right: 75 µmol photons m^{-2} s^{-1}. **A.** Thallus size quantification time course over the course of 57 days; data are mean +/- SE (n=6). **B.** Area under the curve (AUC) per plant over 57 days of experiment; statistics were performed using pairwise wilcox test and p-values were corrected using the bonferroni method (n=6). **C.** Thallus size at 57-days old (n=6); statistics were performed using Welch's ANOVA and p-values were corrected using the Games-Howell post-hoc method. **D.** Gemma cup number per genotype (n=6); statistics were performed using Kruskal-Wallis test and p-values were corrected using the Dunn method (BH-adjusted) at 50 µmol photons m^{-2} s^{-1}, or Welch's ANOVA and p-value correction by Games-Howell post-hoc method at 75 and 100 µmol photons m^{-2} s^{-1}. **E.** Mean grey value of thallus-area (n=6); statistics were performed using Welch's ANOVA and p-value correction by Games-Howell post-hoc method. **F.** Relative chlorophyll a (top) and chlorophyll b (bottom) absorption. Combined results of two independent experiments are shown (n=6); statistics were performed using Welch's ANOVA and p-value correction by Games-Howell post-hoc method. Two independent repetitions of the whole experiment were performed. For the violin plots, the box indicates the middle 50 % of the data, the line indicates the median value (50th percentile), the bottom of the box represents the 25th percentile (Q1) the top of the box represents the 75th percentile (Q3) and the whiskers represent the interval between minimal and maximal values. Violin plot was generated using the Plöttr Toolbox (https://evompmi.github.io/plottr/).

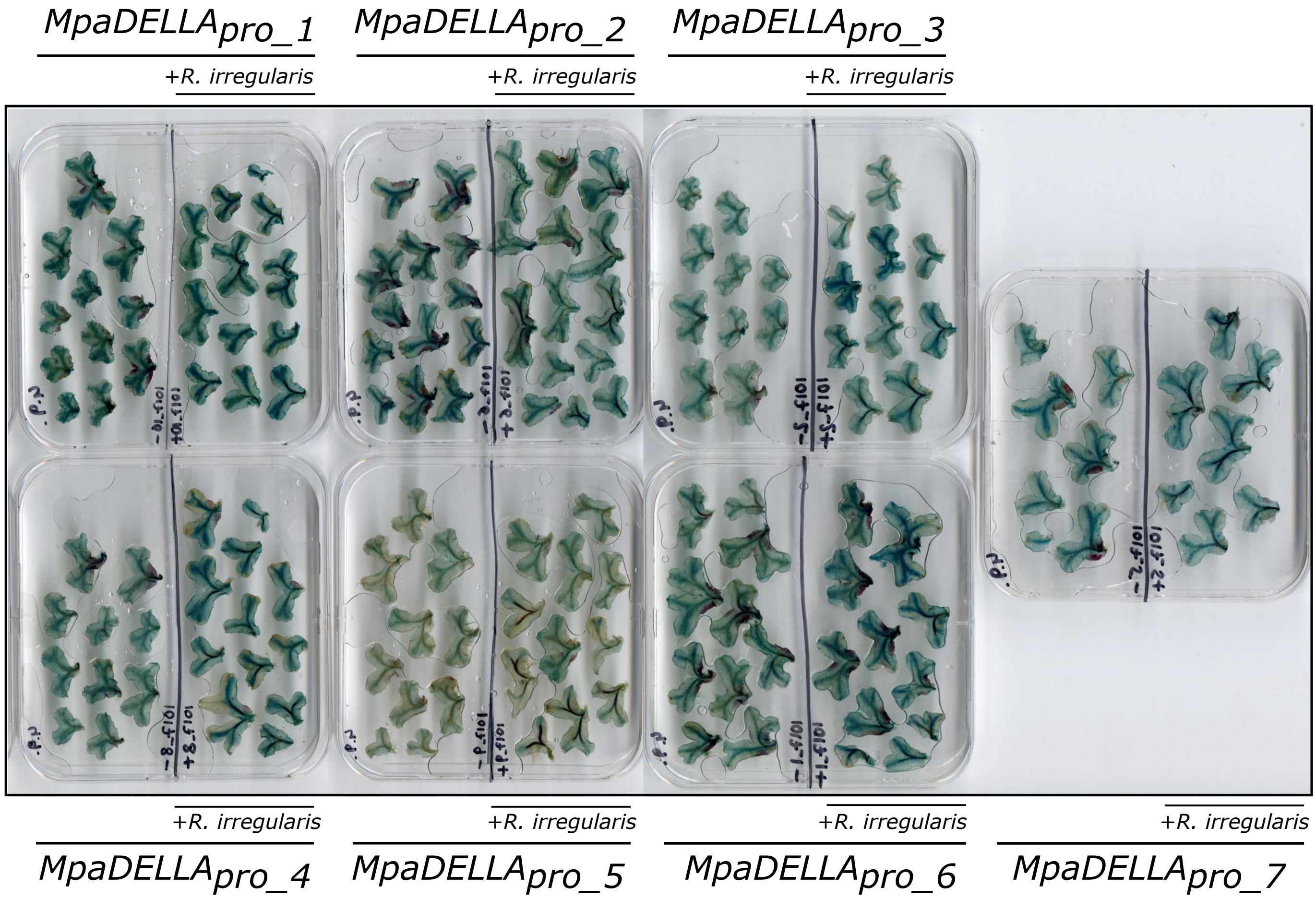

MpaDELLApro_1
+R. irregularis
MpaDELLApro_2
+R. irregularis
MpaDELLApro_3
+R. irregularis
+R. irregularis
MpaDELLApro_4
+R. irregularis
MpaDELLApro_5
+R. irregularis
MpaDELLApro_6
+R. irregularis
MpaDELLApro_7

Fig S4. Overview of Mpa*DELLA* expression profiles in absence or presence of *R. irregularis*. Overview of promoter-GUS reporter expression in seven independent proMpa*DELLA:GUS* lines at 6 weeks post inoculation in absence (left) or presence (right) of *R. irregularis*.

A

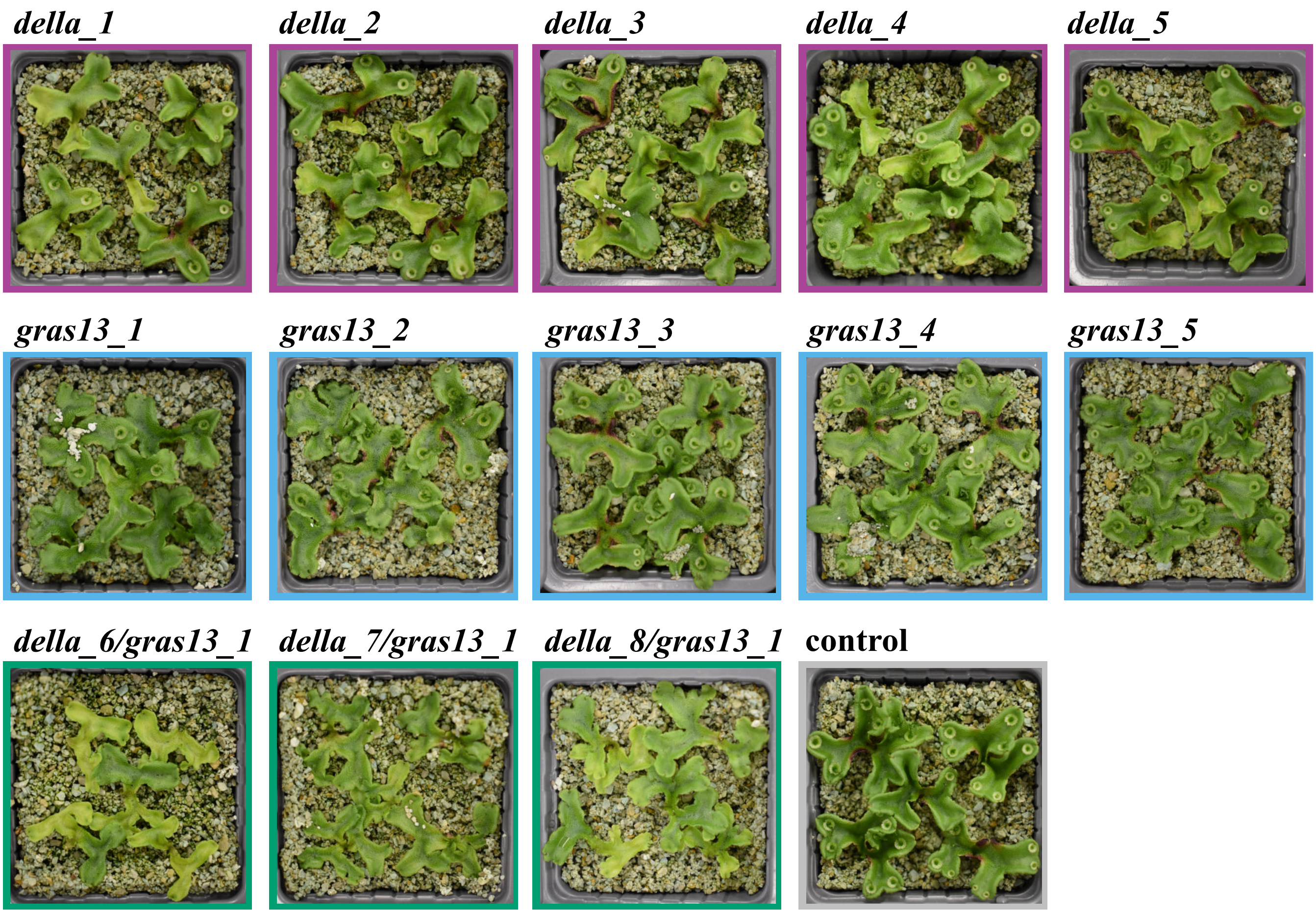

della_1
della_2
della_3
della_4
della_5
gras13_1
gras13_2
gras13_3
gras13_4
gras13_5
della_6/gras13_1
della_7/gras13_1
della_8/gras13_1
control

B

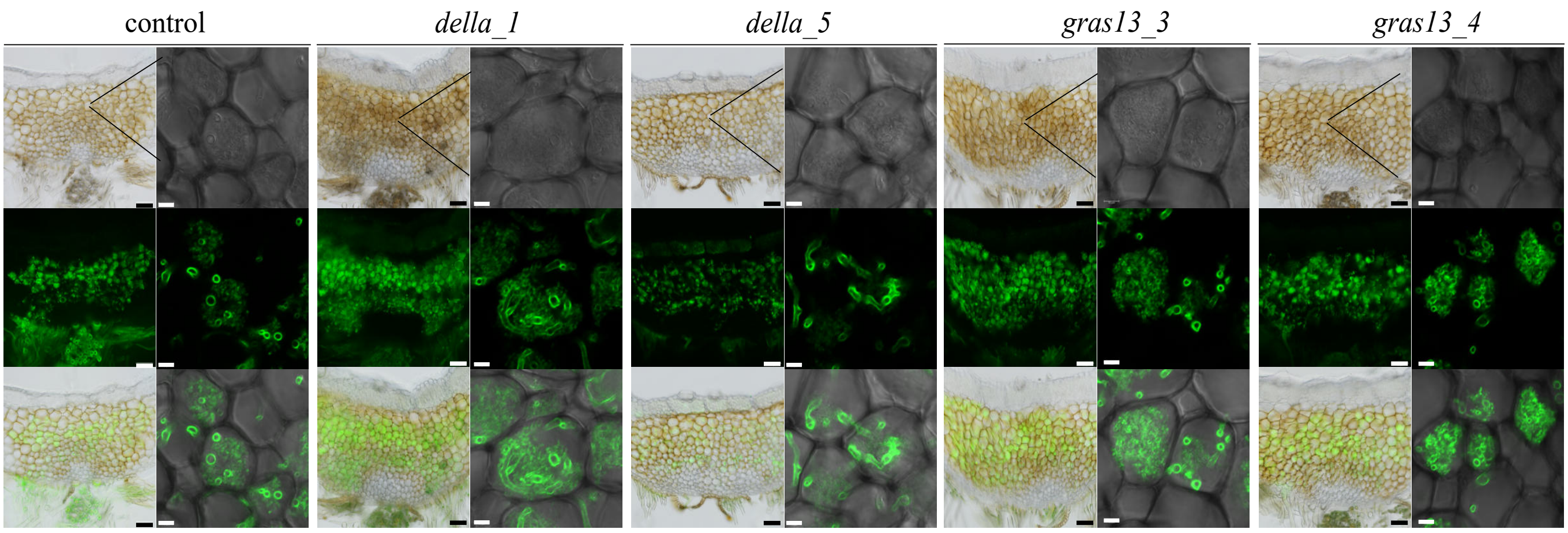

control
della_1
della_5
gras13_3
gras13_4

Fig S5. Macroscopic and microscopic phenotypes of Mpa*della*, Mpa*gras13* and Mpa*della/gras13* mutants. A. Representative pictures of control plants transformed with an empty vector, Mpa*della/gras13*, Mpa*della* and Mpa*gras13* plants inoculated with *R. irregularis* at 6 weeks post inoculation. **B.** Representative microscopic images of control plants transformed with an empty vector and Mpa*della/gras13* double mutants inoculated with *R. irregularis* at 6 weeks post inoculation. Green channel: WGA-Alexafluor 488 staining of fungal structures. Scale bar 100 μM or 10 μM.

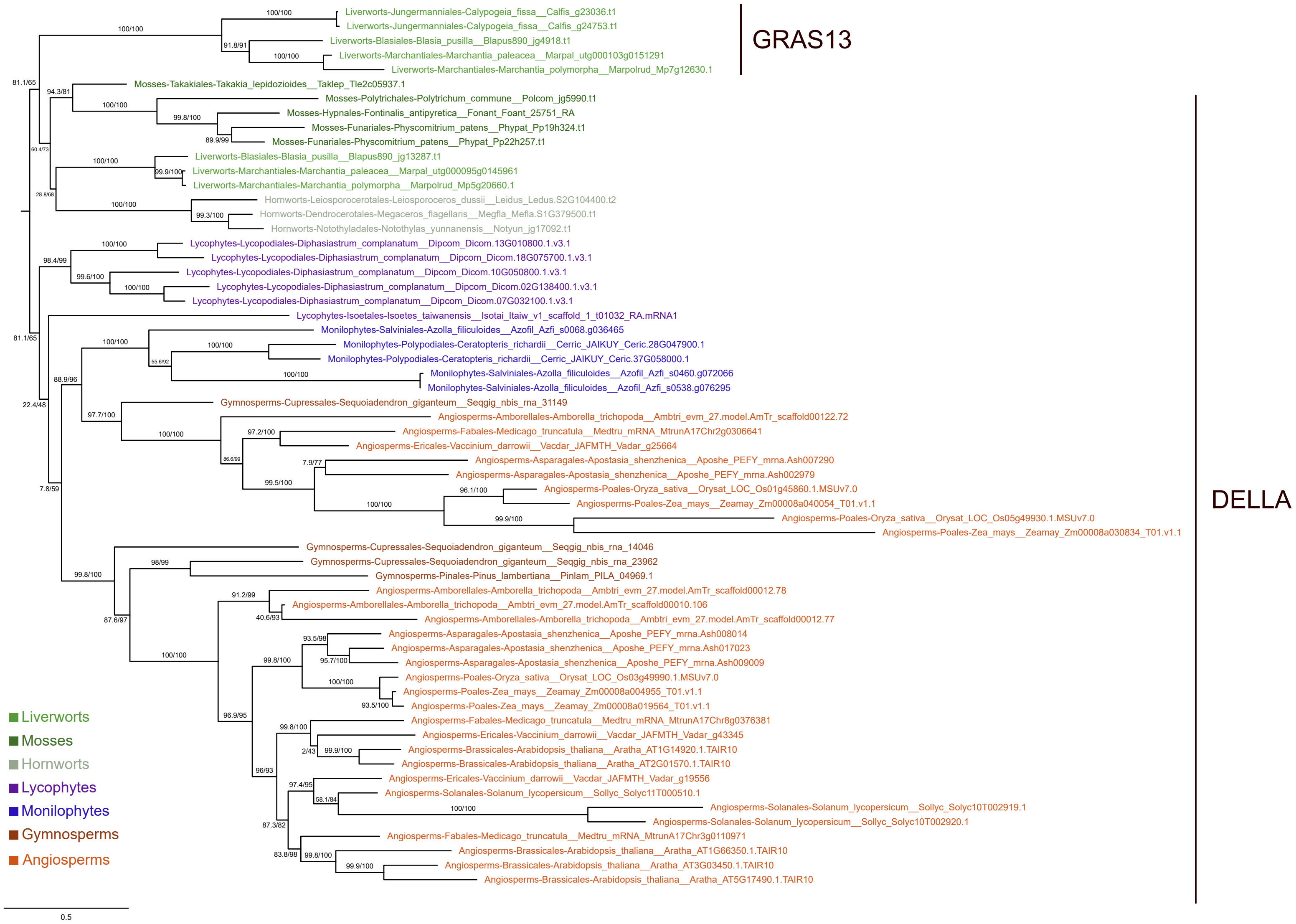
GRAS13
DELLA
Liverworts-Jungermanniales-Calypogeia_fissa__Calfis_g23036.t1
Liverworts-Jungermanniales-Calypogeia_fissa__Calfis_g24753.t1
Liverworts-Blasiales-Blasia_pusilla__Blapus890_jg4918.t1
Liverworts-Marchantiales-Marchantia_paleacea__Marpal_utg000103g0151291
Liverworts-Marchantiales-Marchantia_polymorpha__Marpolrud_Mp7g12630.1
Mosses-Takakiales-Takakia_lepidozioides__Taklep_Tle2c05937.1
Mosses-Polytrichales-Polytrichum_commune__Polcom_jg5990.t1
Mosses-Hypnales-Fontinalis_antipyretica__Fonant_Foant_25751_RA
Mosses-Funariales-Physcomitrium_patens__Phypat_Pp19h324.t1
Mosses-Funariales-Physcomitrium_patens__Phypat_Pp22h257.t1
Liverworts-Blasiales-Blasia_pusilla__Blapus890_jg13287.t1
Liverworts-Marchantiales-Marchantia_paleacea__Marpal_utg000095g0145961
Liverworts-Marchantiales-Marchantia_polymorpha__Marpolrud_Mp5g20660.1
Hornworts-Leiosporocerotales-Leiosporoceros_dussii__Leidus_Ledus.S2G104400.t2
Hornworts-Dendrocerotales-Megaceros_flagellaris__Megfla_Mefla.S1G379500.t1
Hornworts-Notothyladales-Notothylas_yunnanensis__Notyun_jg17092.t1
Lycophytes-Lycopodiales-Diphasiastrum_complanatum__Dipcom_Dicom.13G010800.1.v3.1
Lycophytes-Lycopodiales-Diphasiastrum_complanatum__Dipcom_Dicom.18G075700.1.v3.1
Lycophytes-Lycopodiales-Diphasiastrum_complanatum__Dipcom_Dicom.10G050800.1.v3.1
Lycophytes-Lycopodiales-Diphasiastrum_complanatum__Dipcom_Dicom.02G138400.1.v3.1
Lycophytes-Lycopodiales-Diphasiastrum_complanatum__Dipcom_Dicom.07G032100.1.v3.1
Lycophytes-Isoetales-Isoetes_taiwanensis__Isotai_Itaiw_v1_scaffold_1_t01032_RA.mRNA1
Monilophytes-Salviniales-Azolla_filiculoides__Azofil_Azfi_s0068.g036465
Monilophytes-Polypodiales-Ceratopteris_richardii__Cerric_JAIKUY_Ceric.28G047900.1
Monilophytes-Polypodiales-Ceratopteris_richardii__Cerric_JAIKUY_Ceric.37G058000.1
Monilophytes-Salviniales-Azolla_filiculoides__Azofil_Azfi_s0460.g072066
Monilophytes-Salviniales-Azolla_filiculoides__Azofil_Azfi_s0538.g076295
Gymnosperms-Cupressales-Sequoiadendron_giganteum__Seqgig_nbis_rna_31149
Angiosperms-Amborellales-Amborella_trichopoda__Ambtri_evm_27.model.AmTr_scaffold00122.72
Angiosperms-Fabales-Medicago_truncatula__Medtru_mRNA_MtrunA17Chr2g0306641
Angiosperms-Ericales-Vaccinium_darrowii__Vacdar_JAFMTH_Vadar_g25664
Angiosperms-Asparagales-Apostasia_shenzhenica__Aposhe_PEFY_mrna.Ash007290
Angiosperms-Asparagales-Apostasia_shenzhenica__Aposhe_PEFY_mrna.Ash002979
Angiosperms-Poales-Oryza_sativa__Orysat_LOC_Os01g45860.1.MSUv7.0
Angiosperms-Poales-Zea_mays__Zeamay_Zm00008a040054_T01.v1.1
Angiosperms-Poales-Oryza_sativa__Orysat_LOC_Os05g49930.1.MSUv7.0
Angiosperms-Poales-Zea_mays__Zeamay_Zm00008a030834_T01.v1.1
Gymnosperms-Cupressales-Sequoiadendron_giganteum__Seqgig_nbis_rna_14046
Gymnosperms-Cupressales-Sequoiadendron_giganteum__Seqgig_nbis_rna_23962
Gymnosperms-Pinales-Pinus_lambertiana__Pinlam_PILA_04969.1
Angiosperms-Amborellales-Amborella_trichopoda__Ambtri_evm_27.model.AmTr_scaffold00012.78
Angiosperms-Amborellales-Amborella_trichopoda__Ambtri_evm_27.model.AmTr_scaffold00010.106
Angiosperms-Amborellales-Amborella_trichopoda__Ambtri_evm_27.model.AmTr_scaffold00012.77
Angiosperms-Asparagales-Apostasia_shenzhenica__Aposhe_PEFY_mrna.Ash008014
Angiosperms-Asparagales-Apostasia_shenzhenica__Aposhe_PEFY_mrna.Ash017023
Angiosperms-Asparagales-Apostasia_shenzhenica__Aposhe_PEFY_mrna.Ash009009
Angiosperms-Poales-Oryza_sativa__Orysat_LOC_Os03g49990.1.MSUv7.0
Angiosperms-Poales-Zea_mays__Zeamay_Zm00008a004955_T01.v1.1
Angiosperms-Poales-Zea_mays__Zeamay_Zm00008a019564_T01.v1.1
Angiosperms-Fabales-Medicago_truncatula__Medtru_mRNA_MtrunA17Chr8g0376381
Angiosperms-Ericales-Vaccinium_darrowii__Vacdar_JAFMTH_Vadar_g43345
Angiosperms-Brassicales-Arabidopsis_thaliana__Aratha_AT1G14920.1.TAIR10
Angiosperms-Brassicales-Arabidopsis_thaliana__Aratha_AT2G01570.1.TAIR10
Angiosperms-Ericales-Vaccinium_darrowii__Vacdar_JAFMTH_Vadar_g19556
Angiosperms-Solanales-Solanum_lycopersicum__Sollyc_Solyc11T000510.1
Angiosperms-Solanales-Solanum_lycopersicum__Sollyc_Solyc10T002919.1
Angiosperms-Solanales-Solanum_lycopersicum__Sollyc_Solyc10T002920.1
Angiosperms-Fabales-Medicago_truncatula__Medtru_mRNA_MtrunA17Chr3g0110971
Angiosperms-Brassicales-Arabidopsis_thaliana__Aratha_AT1G66350.1.TAIR10
Angiosperms-Brassicales-Arabidopsis_thaliana__Aratha_AT3G03450.1.TAIR10
Angiosperms-Brassicales-Arabidopsis_thaliana__Aratha_AT5G17490.1.TAIR10
Liverworts
Mosses
Hornworts
Lycophytes
Monilophytes
Gymnosperms
Angiosperms
0.5

Fig S6. Phylogenetic tree of DELLA. Maximum-likelihood tree estimated from amino acid sequences of selected land plant species (model of substitution: JTT+F+I+G4, lnL: -40062.661). Branch support values are shown next to branches (SH-like test/ultrafast bootstrap).

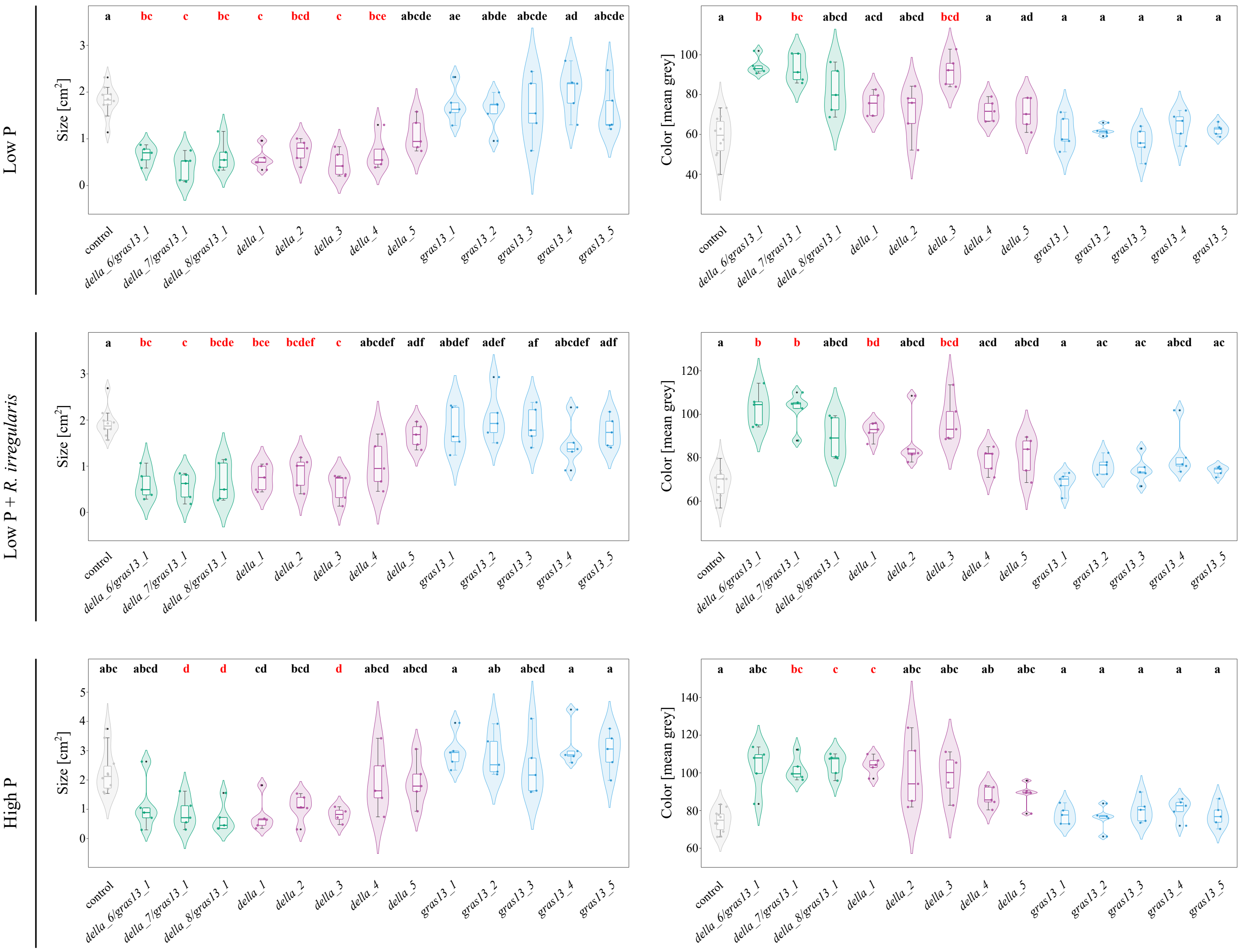
Low P
Size [cm^2]
a bc c bc c bcd c bce abcde ae abde abcde ad abcde
control della_6/gras13_1 della_7/gras13_1 della_8/gras13_1 della_1 della_2 della_3 della_4 della_5 gras13_1 gras13_2 gras13_3 gras13_4 gras13_5
Color [mean grey]
a b bc abcd acd abcd bcd a ad a a a a a
control della_6/gras13_1 della_7/gras13_1 della_8/gras13_1 della_1 della_2 della_3 della_4 della_5 gras13_1 gras13_2 gras13_3 gras13_4 gras13_5
Low P + R. irregularis
Size [cm^2]
a bc c bcde bce bcdef c abcdef adf abdef adef af abcdef adf
control della_6/gras13_1 della_7/gras13_1 della_8/gras13_1 della_1 della_2 della_3 della_4 della_5 gras13_1 gras13_2 gras13_3 gras13_4 gras13_5
Color [mean grey]
a b b abcd bd abcd bcd acd abcd a ac ac abcd ac
control della_6/gras13_1 della_7/gras13_1 della_8/gras13_1 della_1 della_2 della_3 della_4 della_5 gras13_1 gras13_2 gras13_3 gras13_4 gras13_5
High P
Size [cm^2]
abc abcd d d cd bcd d abcd abcd a ab abcd a a
control della_6/gras13_1 della_7/gras13_1 della_8/gras13_1 della_1 della_2 della_3 della_4 della_5 gras13_1 gras13_2 gras13_3 gras13_4 gras13_5
Color [mean grey]
a abc bc c c abc abc ab abc a a a a a
control della_6/gras13_1 della_7/gras13_1 della_8/gras13_1 della_1 della_2 della_3 della_4 della_5 gras13_1 gras13_2 gras13_3 gras13_4 gras13_5

Fig S7. Increased phosphate supply does not fully rescue the developmental phenotypes of Mpa*della* and Mpa*della/gras13* mutants. *Marchantia paleacea* plants transformed with an empty vector (control), Mpa*della*, Mpa*gras13* and Mpa*della/gras13* mutant plants were grown at a light intensity of 65 µmol photons m^{-2} s^{-1} under different nutritional conditions (‘Low P’: Long Ashton solution with 15 µM phosphate, ‘Low P + *R. irregularis*’: Long Ashton solution with 15 µM phosphate and inoculated with 250 spores of *R. irregularis* per plant, ‘High P’: Long Ashton solution with 2.5 mM phosphate). Plant growth and green coloration were quantified after 48 days (5 weeks post inoculation; n = 5). For the violin plots, the box indicates the middle 50 % of the data, the line indicates the median value (50th percentile), the bottom of the box represents the 25th percentile (Q1) the top of the box represents the 75th percentile (Q3) and the whiskers represent the interval between minimal and maximal values. Violin plot was generated using the Plöttr Toolbox (https://evompmi.github.io/plottr/).

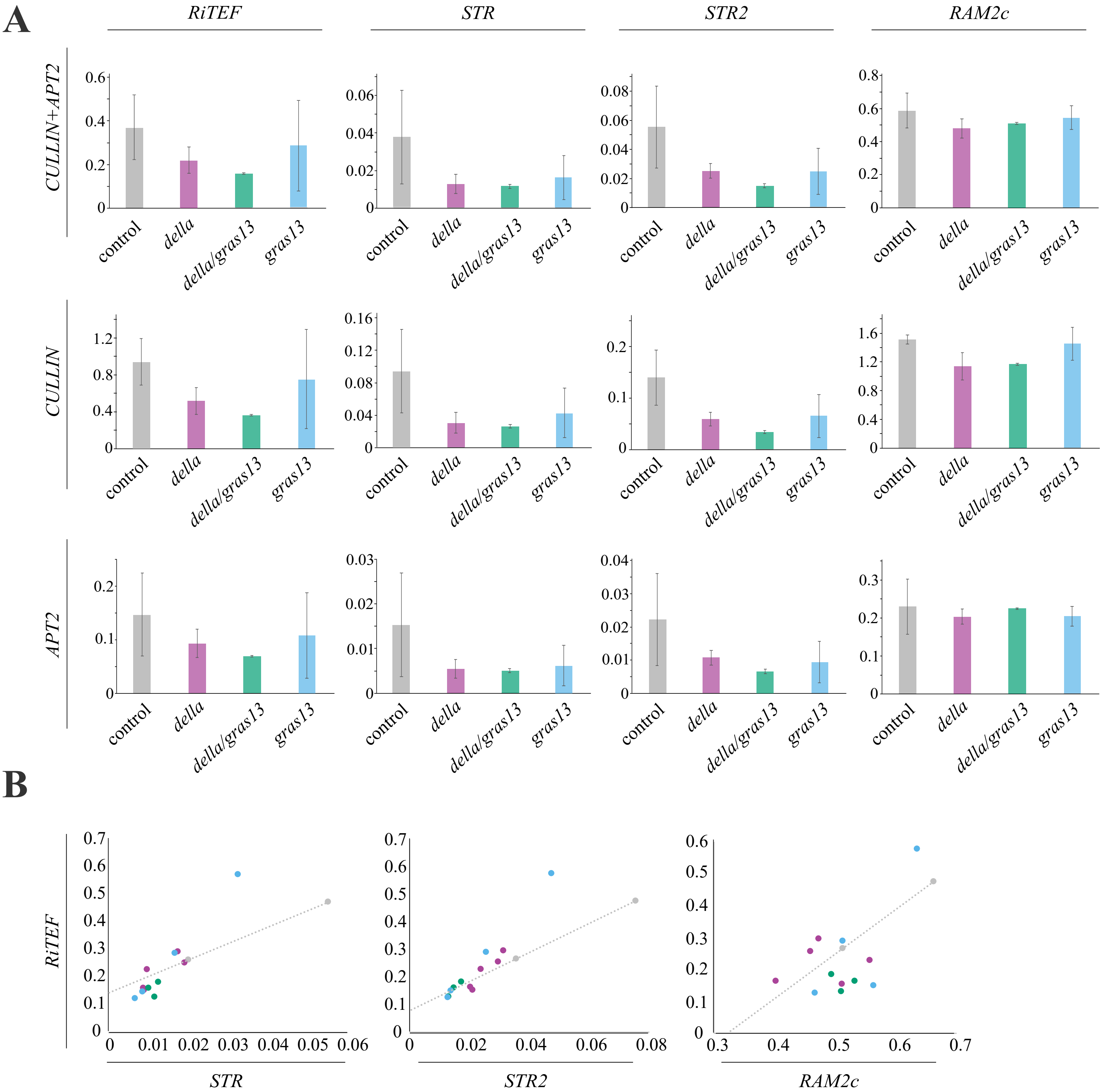
A
RiTEF
STR
STR2
RAM2c
CULLIN+APT2
CULLIN
APT2
control
della
della/gras13
gras13
B
RiTEF
STR
STR2
RAM2c

Fig S8. Symbiosis marker gene expression in Mpa*della*, Mpa*gras13* and Mpa*della/gras13* plants. A. Relative expression of Ri*TEF* was quantified to assess fungal colonization and symbiosis marker genes Mpa*STR*, Mpa*STR2* and Mpa*RAM2c* were quantified in all samples (n = five plants per sample). Independent lines and loss-of-function mutant alleles were combined later for the analysis. Relative expression values were calculated using the reference genes Mpa*CULLIN* and Mpa*APT2* (data correspond to 2-ΔCT with ΔCT = CTgene of interest-CTaverageCTCULLIN + APT2) or Mpa*CULLIN* and Mpa*APT2* alone. Data are mean +/- SD. No statistically significant differences were found. B. Comparison of symbiosis marker gene expression depending on fungal load as quantified by relative expression of Ri*TEF*. Each dot corresponds to one sample with n = 5 plants per sample. Grey: control plants, magenta: Mpa*della* single mutants, turquoise: Mpa*gras13* single mutants, green: Mpa*della/gras13* double mutants.

Table S1. Plasmids, guideRNA sequences and qPCR primers.

Table S2. Supplementary data for *Marchantia paleacea della* experiments at different light regimes.

Table S3. List of land plant species genomes used for the phylogenetic analyses.

References

Vernié, T., Rich, M., Pellen, T., Teyssier, E., Garrigues, V., Chauderon, L., Medioni, L., Van Beveren, F., Libourel, C., Keller, J., Girou, C., Lefort, C., Le Ru, A., Martinez, Y., Reinhardt, D., Kodama, K., Shimazaki, S., Morel, P., Kyozuka, J., Mbengue, M., Vandenbussche, M., Delaux, P.-M., 2025. Conservation of symbiotic signaling since the most recent common ancestor of land plants. Proc. Natl. Acad. Sci. U.S.A. 122, e2408539121. https://doi.org/10.1073/pnas.2408539121

Engler C., Youles M., Gruetzner R., Ehnert TM., Werner S., Jones JD., Patron NJ., Marillonnet S., 2014. A golden gate modular cloning toolbox for plants. ACS Synth Biol. 3(11):839-43. https://doi.org/10.1021/sb4001504
